# Social marketing and happiness in employment. Evidences from Glassdoor

**DOI:** 10.1186/s40359-024-01882-8

**Published:** 2024-08-17

**Authors:** Araceli Galiano-Coronil, Sofía Blanco-Moreno, Luis Bayardo Tobar-Pesantez

**Affiliations:** 1 University of Cádiz, Jerez de la Frontera, Spain; 2 University of León, León, Spain; 3 Politécnica Salesiana University, Cuenca, Ecuador

**Keywords:** Social marketing, Happiness management, Laboral satisfaction, Glassdoor, Text mining

## Abstract

**Background:**

With the increasing role of the Internet and social media, there are more significant opportunities for employees to express their opinions about the companies they work for more directly. A recognized job review website is Glassdoor.com, which collects employees’ opinions anonymously and the scores they give to companies. This descriptive study analyzes the assessment that employees give to companies by studying the advantages in their comments from the perspective of Happiness Management and Social Marketing. In this sense, this research aims to analyze how the main benefits offered by companies, are linked to the happiness of employees and to the actions of social marketing that companies develop affect the general satisfaction of employees.

**Methods:**

This study has used in the worker comments, text mining, and inferential analysis techniques. The sample was divided into two blocks, with comments that refer to issues about social marketing and happiness. In each one, an inferential analysis was carried out using the Student’s T-test. This analysis allowed us to identify, in each sample of comments, in which advantages the differences in the mean ratings were significant depending on whether they were mentioned.

**Results:**

The main results indicate that social marketing and happiness are linked to the advantages employees comment on in reviews on Glassdoor. Significant differences exist in the average ratings of certain advantages depending on whether they are mentioned or not in the comments. Likewise, the differentiation between comments on social marketing and happiness offers scientific evidence of the most valued advantages in each cluster. Specifically, the advantages grouped into the following dimensions are working conditions, company image, and social relations.

**Discussion:**

This research contributes to happiness management theories by empirically demonstrating how positive work environments enhance productivity, loyalty, and creativity. These insights show how leadership quality, work-life balance, and recognition contribute to workplace happiness, enhancing productivity, loyalty, and creativity. Such feedback aids job seekers in making informed decisions, helps companies improve practices and attract talent, and provides researchers with valuable data on employment trends and corporate culture’s effect on employee well-being.

## Introduction

In the evolving landscape of Industry 5.0, where human-centric technology and automation converge, workplace dynamics are undergoing a significant transformation. This era not only emphasizes the integration of advanced technologies into the fabric of daily work but also places a renewed focus on the human elements of creativity, ethics, and happiness. As we navigate through this new industrial revolution, understanding the factors that contribute to employee happiness and job satisfaction becomes paramount [1].

The concept of the happiness economy, rooted in the belief that employee well-being directly contributes to organizational success, is gaining traction among scholars and practitioners alike [2]. Research has consistently shown that happy employees exhibit higher levels of productivity, creativity, and loyalty, which are critical drivers of innovation and competitive advantage in today’s fast-paced business environment [2, 3]. In the context of Industry 5.0, where the boundaries between technology and human capabilities are increasingly blurred, fostering an environment that prioritizes employee happiness is not just a moral imperative but a strategic necessity.

Online platforms like Glassdoor offer a wealth of data on employee experiences and perceptions, providing insights into the factors that influence job satisfaction and happiness [4]. These platforms, characterized by their user-generated content, offer a candid look at the internal workings of organizations, as seen through the eyes of current and former employees [5]. Analyzing reviews on Glassdoor can reveal patterns and trends related to job satisfaction, employee engagement, and the effectiveness of management practices [6]. Furthermore, such analysis can help identify the job benefits most valued by employees, offering a roadmap for organizations looking to enhance their value proposition to attract and retain top talent. The relationship between job benefits and employee happiness is a critical area of inquiry in the happiness economy [7]. Benefits such as flexible working arrangements, health and wellness programs, and opportunities for professional development have been linked to higher levels of job satisfaction and overall well-being [5]. In Industry 5.0, where the nature of work is continually evolving, the ability to offer innovative and meaningful benefits is increasingly important [8]. Organizations that successfully align their benefits packages with the values and needs of their workforce can create a more engaged, motivated, and happy employee base, driving innovation and success in the process [9].

This paper will delve into the methodologies for analyzing employee reviews on the Glassdoor platform, discussing the challenges and opportunities associated with mining this rich source of qualitative data [10]. It will also examine the role of advanced analytics in extracting actionable insights from employee feedback, a capability that is particularly relevant in the data-driven environment of Industry 5.0 [11]. By leveraging these technologies, organizations can gain a deeper understanding of employee sentiment, identify areas for improvement, and tailor their strategies to enhance job satisfaction and happiness. Moreover, the paper will explore the implications of job benefits on employee happiness within the context of Industry 5.0 given that some companies say they care about the well-being and “happiness” of their employees, but there is not enough scientific support [12]. It will argue that in an era characterized by rapid technological advancement and changing work paradigms, traditional benefits packages may no longer suffice. Instead, organizations must think creatively and holistically about how to support their employees’ well-being, considering factors such as work-life balance, mental health, and lifelong learning opportunities [13].

As we move further into the era of Industry 5.0, the link between innovation and the happiness economy becomes increasingly evident [14]. By analyzing employee reviews on platforms like Glassdoor, organizations can gain valuable insights into the factors that contribute to job satisfaction and happiness [15]. Furthermore, by understanding and addressing the evolving needs of their workforce, companies can leverage job benefits as a powerful tool for fostering a happy, productive, and innovative workplace [16]. This paper aims to contribute to the growing body of literature on the happiness economy in Industry 5.0, offering a comprehensive analysis of the role of employee feedback and job benefits in shaping the future of work. As indicated, analyzing happiness and job satisfaction through employee reviews on online platforms like Glassdoor is crucial for several reasons, impacting both the workforce and the organizations they belong to [17]. This analysis, encompassing job benefits and their relationship with job happiness and social marketing, offers a multifaceted understanding of the current work environment and employee well-being.

In the context of Industry 5.0, the significance of social marketing in enhancing employee happiness cannot be overstated. Social marketing, with its core in influencing behaviors for social good, extends into the workplace by shaping positive organizational cultures and promoting well-being. It plays a pivotal role in communicating the organization’s values and commitment to employee happiness, which is crucial in attracting and retaining top talent in a competitive landscape. Effective social marketing strategies not only spotlight the organization’s dedication to its employees’ well-being but also foster a sense of belonging and community among workers. By actively engaging in social marketing, companies can elevate their employer brand, making them more appealing to prospective and current employees. This approach aligns with the principles of the happiness economy, where the well-being of employees is seen as integral to organizational success. Through social marketing, organizations can effectively showcase their innovative benefits, work-life balance initiatives, and commitment to creating a supportive and happy workplace, thereby enhancing job satisfaction and overall organizational performance.

Intrapreneurship is essential for fostering innovation and enhancing employee satisfaction. Galván-Vela et al. [18] highlight how intrapreneurial activities contribute to a positive work culture and job satisfaction. Encouraging intrapreneurship boosts morale, creativity, and overall workplace happiness.

Given the importance of managing happiness in the work environment and its social, economic and political implications, this research aims to analyze how the main benefits offered by companies, are linked to the happiness of employees, and to the actions of social marketing that companies develop affects the general satisfaction of employees. Likewise, given that the benefits offered differ between the different sectors of the companies since there are sectors in which teleworking cannot be established, for example, the differences produced between the sectors are delved into. For all these reasons, given that there are studies that analyze the benefits offered by companies to their employees, through comment platforms such as Glassdoor, but none from the perspective provided by the results of the semantic network analysis, this research has been developed. This descriptive study analyzes the assessment that employees give to companies by studying the advantages in their comments from the perspective of Happiness Management and Social Marketing. To do this, first of all, the groups of advantages have been identified whose presence in the comments makes a difference in the average ratings compared to their absence. Secondly, the analysis of the groups of advantages detected was carried out in-depth to verify which advantages, more specifically, contribute to the formation of clusters that generate certain patterns of communication and behavior.

This article is structured as follows. In the first section, a theoretical framework is proposed that explains the importance of the benefits offered by companies in the satisfaction and happiness of employees. In the second section, we describe our sample and methods. The third section is devoted to the presentation of the results and the fourth section discusses the results and their implications for theory and research. We then conclude by presenting the study’s main limitations, along with future research directions.

## Theoretical background

### Understanding employee perspectives and the link with happiness management

To maintain organizational effectiveness and achieve high performance, companies need to develop and maintain happy environments [19]. Analyzing employee perspectives is crucial for enhancing work happiness in companies, not only because employee behaviors in the workplace affect organizations and customers, but also employees’ perceptions of the workplace, such as their job satisfaction reflected in online reviews from other employees [20]. Knowing these employee perspectives provides direct insights into the workforce’s needs and expectations. Employees are the backbone of any organization, and understanding their viewpoints helps in creating a work environment that caters to their well-being and job satisfaction. This understanding is key to fostering a positive and productive workplace [21]. Employee feedback highlights areas for improvement too. Whether it’s about work conditions, management styles, or company policies, employee perspectives can pinpoint specific issues that might be hindering job satisfaction [22]. Addressing these issues not only improves the work environment but also demonstrates that the company values its employees’ opinions, thereby boosting morale and loyalty [23].

Analyzing employee perspectives aids in tailoring benefits and incentives. Different employees might value different aspects of their jobs, such as flexible working hours, professional development opportunities, or wellness programs [5]. Understanding these preferences allows companies to offer more meaningful and effective benefits, directly contributing to increased job happiness. And also, it promotes a culture of openness and trust. When employees feel heard and see their feedback leading to positive changes, it builds trust in the organization. This trust is fundamental for a harmonious work environment where employees feel valued and happy [24]. For all of this, analyzing employee perspectives is a vital step towards creating a more fulfilling, productive, and happy workplace. Analyzing these perspectives indicated by employees also allows us to better understand what makes employees happy, something that is essential for improving a company’s recruitment strategy [25].

The role of cultural factors in shaping employee happiness cannot be overlooked. Sanagustín-Fons et al. [26] explored the interplay between happiness and cultural tourism, emphasizing the perspective of civil participation. Their findings indicate that cultural engagement and community involvement are significant predictors of happiness. This underscores the importance of fostering a workplace environment that values cultural participation and community-oriented activities, which can enhance employees’ overall well-being and job satisfaction.

It enables the creation of a compelling employer value proposition. When a company knows what drives employee satisfaction, it can highlight these aspects in its recruitment messaging, attracting candidates who are more likely to be fulfilled and engaged in their roles [27]. Furthermore, aligning recruitment strategies with factors that contribute to employee happiness can significantly enhance the quality of hires. Candidates are increasingly looking for workplaces that prioritize not just financial compensation but also aspects like work-life balance, career development opportunities, and positive company culture [28]. By showcasing a commitment to these values, companies can attract talent that resonates with their organizational ethos. Moreover, understanding employee happiness helps in reducing turnover [29]. When new hires find that their expectations, shaped by the recruitment process, match their actual job experience, they are more likely to stay with the company. This alignment reduces the costs and disruptions associated with high staff turnover.

A recruitment strategy informed by employee happiness insights reflects a company’s commitment to its workforce’s well-being. This not only enhances the company’s reputation in the job market but also fosters a positive internal culture, making it an employer of choice for top talent. In essence, knowing what makes employees happy is a strategic tool for attracting and retaining the right people, crucial for the long-term success of any organization [30]. Several studies highlight the importance of analyzing online employee reviews for insights into employee satisfaction, organizational perception, and the predictive value of such reviews on firm performance. On the one hand, some authors investigated the informational value of online employee reviews and their predictive ability on firm financial performance [31]. The study found that employee online reviews have informational value and can predict firm performance, indicating that integrating structured and unstructured data can enhance decision support systems. They suggest further exploration into how different sectors utilize employee feedback for strategic advantages. Similarly, Symitsi et al. [31] explored the value of online employee reviews from various perspectives, including their impact on firm performance. They confirmed the significant predictive value of employee reviews on firm performance, advocating for the integration of big data in managerial decisions. And they point towards leveraging big data for competitive advantage and enhancing firm strategies. On the other hand, Sekar et al. [32] investigated the impact of employees’ perceptions of their organization on overall satisfaction using online reviews. They found that positive sentiments in reviews significantly affect overall employee satisfaction, with skill development being a strong predictor of satisfaction. Besides, Koncar & Helic [33] explored and predicted employee satisfaction using a novel dataset of two million online employer reviews, and they identified the number of benefits received and employment status as the most predictive factors for employee satisfaction, with less emphasis on employee position, suggesting employers use these insights to correct biases in assessing their reviews and improve satisfaction. A similar analysis was carried out by Querbach et al. [5], but focusing on aspects of social well-being.

### Job benefits and happiness management through glassdoor

There is a direct correlation between job benefits and employee happiness [34]. Benefits like health insurance, retirement plans, paid time off, and flexible working hours are often cited in reviews as key factors in job satisfaction [35]. While salary is important, reviews often highlight the value of non-monetary benefits [36]. For instance, work-life balance, professional development opportunities, and positive work culture are frequently mentioned concerning job satisfaction [37].

Analyzing platforms like Glassdoor to understand the job benefits that contribute to employee happiness is crucial. These platforms offer unfiltered, real-world insights from a diverse range of employees. Unlike controlled surveys or internal feedback mechanisms, Glassdoor reviews often provide candid opinions about what benefits truly impact employee satisfaction. This raw feedback is invaluable for understanding the actual effectiveness of various benefits [30]. By examining these platforms, companies can identify trends and patterns in employee preferences. For instance, while traditional benefits like health insurance and retirement plans are consistently valued, there may be a growing appreciation for flexible working arrangements, mental health support, or professional development opportunities [28]. Understanding these evolving preferences helps companies stay ahead in offering competitive and relevant benefits. Analyzing employee feedback on platforms like Glassdoor assists in tailoring benefits packages to meet the specific needs of the workforce. This customization is key to enhancing job satisfaction and overall happiness [28]. When employees see that their employer is responsive to their needs and values their well-being, it fosters a positive work environment and strengthens employee loyalty. This analysis can also serve as a benchmarking tool. Companies can compare their benefits with competitors and industry standards, ensuring they remain attractive to both current and prospective employees [24]. So, analyzing Glassdoor reviews provides critical insights into the benefits that genuinely contribute to employee happiness, enabling companies to make informed, impactful decisions in their benefits offerings.

Glassdoor provides a deep knowledge about job benefits. Investigating which benefits make employees happier is also important because happy employees are more productive. When employees receive benefits that genuinely meet their needs and preferences, such as flexible working hours, comprehensive health insurance, or opportunities for professional growth, their job satisfaction increases [38]. This satisfaction translates into higher engagement and motivation at work. Happy employees are more likely to be committed, creative, and proactive, contributing positively to their tasks and the overall success of the organization. Therefore, identifying and providing the right benefits is not just about employee well-being; it’s a strategic approach to enhancing workplace productivity and organizational performance. Several studies highlight the importance of analyzing online employee reviews to understand the relationship between job benefits, employee satisfaction, and corporate performance. Coaley [39] explored employee reviews on Glassdoor and Indeed for four Las Vegas hotel/casino corporations to understand the employer-brand benefits. It was identified three main employer-brand benefits from the reviews: functional, economic, and psychological, each appearing as both positive and negative attributes of employment, and it was suggested further research into how these employer-brand benefits impact recruitment and retention strategies. In Brazil, da Silveira [40] investigated the effect of employee satisfaction on corporate performance using online reviews, and found a positive association between overall employee satisfaction and firm performance, especially in dimensions related to culture and career opportunities, while compensation and benefits were less connected. Finally, Zia & Sheikh [41] studied the impact of job satisfaction on employee performance, particularly in the context of Pakistan. They found significant determinants of employee performance related to financial and non-financial rewards and suggested a deeper investigation into the specific aspects of job satisfaction that most strongly influence performance across different industries.

### Social marketing social and laboral happiness

Social marketing is a marketing discipline that, since it emerged at the end of the 1960s, has been configured as an ideal tool to promote behavioral change in a target audience to improve individual well-being and that of citizens [42]. The approach used by social marketing to carry out a behavioral change focuses on discovering the barriers and motivations of the target audience and designing a plan for this purpose using the 4Ps of the marketing mix [43, 44]. Edgar, Boyd, and Palame [45] have suggested that social marketing has more potential to stimulate sustainable behavioral change in target populations than other educational interventions. It is essential in the workplace since he is a happy worker, demonstrated by his behavior by performing better and executing his tasks with greater satisfaction [46]. Likewise, workers present characteristics such as being tolerant and generous and having better problem-solving abilities. In addition, work happiness can also modify the environment of employees to achieve coexistence in a space with a good work environment, encourage collaborative participation and adaptability, and promote creativity and innovation [47].

In the effectiveness of social marketing in the workplace, it must be considered that individual behaviors are integrated into an ecological system in the sense that people carry out their behaviors within a historical, social, cultural, physical, and environmental environment. The behavioral ecological approach derives from the principles of environmental systems, in which everything is seen as interrelated within a complex system. It means that behaviors depend on the interaction between actors, their influences, interactions and reactions, and the co-creation of results [48]. Correct communication between these actors is vital in this context since one of the workplace’s main barriers to achieving this goal is cognitive or the need for information. Fishbein’s expectancy-value model [49] suggests a theoretical approach to rational decision-making. This approach is used when the decision has significant implications and, therefore, much cognitive effort is invested in making this decision. In the workplace, job evaluation is based on its conditions, for example, salary level, job interest, ability to combine work and family obligations, and belief in professional success.

The relevance of social responsibility in sustainable organizations is increasingly evident. Hernández García de Velazco et al. [50] analyzed the social responsibility of sustainable university organizations, highlighting the impact of endogenous capacities on organizational effectiveness. Their study demonstrates that socially responsible practices are integral to creating a supportive and happy work environment. By embedding social responsibility into their core values, organizations can enhance employee satisfaction and loyalty, which are critical for long-term success.

In this sense, the social marketing approach becomes critical because it involves analyzing the barriers and motivations to achieve a particular behavior, such as a positive and happy attitude towards work. Furthermore, attitudes, relationships, feelings of belonging, interpersonal skills, participatory decision-making, and effective communication play a crucial role in the performance and effectiveness of an organization [51]. Resources that allow optimizing the previous factors lead to positive attitudes, which result in pro-social behaviors [52].

Finally, it is important to also mention that companies can generate a better image of themselves through social marketing campaigns [53–55]. For example, employee reviews show positive environments and high job satisfaction [56], thanks to working conditions [57, 58]. In fact, company image is also linked to reputation and culture [59–61].

## Methodology

With the increasing role of the Internet and social media, there are more significant opportunities for employees to express their opinions about the companies they work for more directly. It can even be stated that social networks have weakened companies’ control over their information environment [62]. In this environment, Glassdoor is configured as an ideal platform to study employee opinions, as it allows employees to anonymously review the companies they currently work for or have once worked for and rate their overall satisfaction with these companies from one (low) to five (high) stars. The design of this website reduces bias in its ratings, and its design features support the quality of its ratings [63]. Focusing on Glassdoor, this study analyzes the benefits most valued by workers and delves deeper into the context.

This research uses a descriptive methodology that combines content analysis, inferential analysis, and text mining. Text mining on Glassdoor comments is an area of ​​growing interest that focuses on discovering patterns and insights in unstructured data [64, 65]. However, the effectiveness of different text mining tools in this context has not yet been explored in depth [66]. One potential improvement area is using more complex representations of text content, such as conceptual graphs, to improve the discovery of meaningful patterns, such as that proposed with semantic network analysis [67]. Most studies have been based on primary data collected through the survey. However, research indicates that the external validity of survey-based studies is compromised due to sampling bias [68]. In this sense, employee-generated content on platforms like Glassdoor can be a valuable source of information, as it provides unsupervised information, which is impossible in a questionnaire-based survey method.

Various studies have applied statistical inference with the T-student distribution in data mining, most focusing on clustering, classification, and association models, demonstrating its value. In addition, its application in large samples is also valid due to the Central Limit Theorem, which states that the distribution of sample means will have an approximately normal distribution, regardless of the population from which the samples are drawn. This theorem allows using the student t-test in large samples since it ensures that the distribution of the sample means will be expected, even if the original data are not [69].

Content analysis is widely used for marketing and communication due to its effectiveness and big-volume data accommodation. As a unit of analysis, we have considered the reviews from the employees in Glassdoor, and the variables for codifying have been mentioned in the previous paragraph [70]. The sample was divided into two blocks, one with comments that refer to issues about social marketing and another with comments that prioritize aspects related to happiness. In each of these samples, an inferential analysis was carried out using the Student’s T test, making the comparison level of means more feasible. This analysis allowed us to identify, in each sample of comments, in which advantages the differences in the mean ratings were significant depending on whether they were mentioned. This analysis was also carried out by sector. Once these advantages were detected, we proceeded to delve deeper into the content using text mining. Text mining allows for extracting the relevant information necessary for the study, summarizing, and discovering trends and patterns in textual data. A semantic network analysis, derived from text mining, was used to quantitatively explore the correlation between the keywords obtained [71] and thus obtain a more detailed analysis of the context in which the most valued advantages appear, both in the comments of workers who give importance to social marketing activities, as well as those who prioritize issues related to happiness.

### Variables identification

After reviewing the literature, the aspects most commented on and valued by employees in terms of benefits have been detected. Although non-financial characteristics explain job satisfaction better than monetary rewards among European Union employees, there are various classifications of benefits [72]. On the one hand, Querbach et al. [5] categorized the job benefits enhancing the three main dimensions of well-being, based on the previous work of Grant et al. [73]:

- Physical: on-site cafeteria, food allowance (free company-provided meals), pension scheme, healthcare, company doctor, and parking. They categorized it as job benefits of care.
- Psychological/status: flexible working hours, coaching, home office, vehicle allowance, and company phone. They categorized it as job benefits of status.
- Social well-being: on-site daycare facility/reimbursed daycare, handicapped accessibility, employee events, stock or equity options, and employee discount. They categorized it as job benefits of life quality.

However, this classification ignores aspects valued by other authors, which have been shown to affect happiness management. For example, this classification is omitting aspects such as the importance of salary, social status, contribution to the community, interest, success or support that Friedmann [74] has valued. Omodan et al. [75] analyzed aspects such as attitudes in the work environment, relationships, feelings of belonging, interpersonal skills, participatory decision making and effective communication. Coaley [39] identified three main employer-brand benefits from online reviews: functional, economic, and psychological. Finally, the classification of Querbach et al. [5] does not consider aspects such as career or job promotion, community membership, job opportunities, co-workers, the nature of the work, or relationships with supervisors, as Salas-Vallina & Alegre [76] did. In addition, these authors also provided a list of adjectives for happiness at work, such as bursting, excited, accomplished, time flies, inspiring, happy, proud, challenging, concentrated/immersed, hooked/involved, persistent/constant.

For all this, for this research, an adaptation of the benefits proposed by these five authors has been carried out, which have finally been grouped into seven dimensions of variables: Facilities, Labor conditions, Relationships, Company, Benefits, Social Marketing and Happiness Management.

### Sample and data collection

Glassdoor (https://www.glassdoor.com/) is a website that offers an inside look at jobs and companies through user-generated content. It provides a platform for current and former employees to anonymously submit reviews about their workplaces, including insights into company culture, salaries, benefits, and management practices. Additionally, Glassdoor offers information on job openings, interview questions, and company ratings. This transparency aims to help job seekers make informed decisions about their career paths and for employers to understand how they can improve their workplace environment. Glassdoor has become a valuable resource for both job seekers and companies in the job market ecosystem [77].

For this research, 138,764 reviews have been downloaded, from 136 companies, between the years 2021 and 2023. The sample was selected based on several criteria to ensure a robust and representative dataset. Only companies with more than 10,000 employees were included. This criterion was set to ensure that the reviews represented experiences from large and potentially more structured organizations. Companies from various sectors such as construction, consulting and advisory, consumption, financial sector, industry, computing, advertising and public relations, healthcare, telecommunications, transportation and tourism were selected. This diversity aims to capture a wide range of employee experiences across different industries.

The data were downloaded with the web scraping technique, through the Octoparse version 8 software. This tool allows the downloading of large amounts of data in a structured way, which facilitates its cleaning and analysis by directly obtaining the data ordered by rows (each anonymous review) and columns (characteristics of each review, such as rating, advantages, disadvantages, etc.).

Before this grouping, a classification of each review was carried out based on these previously mentioned variables. For classification, a dictionary of words that have been searched within each review has been prepared.

As it was mentioned, the sample was divided into two blocks, one with reviews that refer to issues about social marketing and another with reviews that prioritize aspects related to happiness. After this classification, the sample was reduced to 1,877 social marketing reviews and 2,163 happiness reviews.

### Network semantic analysis

Dividing conversational texts into groups based on word associations and visualizing them in network form has become a significant achievement in text mining research [78]. The Gephi tool has been used to visualize clustering in complex texts. Gephi is a platform Sebastien Heynmann, and Mathieu Jacomy developed for the interactive visualization and exploration of all types of networks, complex systems, and dynamic and hierarchical graphs. Its functionalities include importing, exporting, manipulating, analyzing, filtering, representing, detecting communities, and exporting large graphs and networks [79, 80]. The following functionalities have been selected to display the network: Results of the analysis of comments linked to social marketing.

The Fruchterman Reingold distribution algorithm, which offers more robust visual and analytical capabilities for clustering [81], has been considered. The words have been structured into communities following the Louvain method developed by Blondel et al. [82]. This community detection method seeks to optimize modularity, a number between − 0.5 and 1 that compares the density of edges inside and outside a community—in theory, optimizing this value iteration by iteration results in the best possible grouping of the nodes of a network. However, going through all possible iterations from nodes to groups is impractical; therefore, different heuristics are used. In the Louvain Method of community detection, small communities are first found by optimizing modularity locally for all nodes. Each small community is associated with a node, which repeats until convergence. In this sense, connections are generated through co-currency; if two words appear in the same publication simultaneously, they are considered semantically related, and the connection that gives rise to the network is produced. Finally, the nodes associated with each word have been differentiated with different colors, using the GePhi option to show the communities on radial axes (Radial Axis Layout), aligning the words of each community to facilitate their understanding.

Finally, the PageRank of each word has been calculated [83], which identifies the relative importance of the word within the network. Unlike simply counting the number of words to which it is related, this measure also gives importance to a word or node as a weighted average of the importance of other words or nodes that connect with it [84]. The size of each node (words that have an ordinary meaning and are represented as a circle in the network) is proportional to its PageRank value, with the value of the largest node being the one with the largest.

## Results and discussion

### Results of the analysis of comments about social marketing

Below are the results corresponding to the quantitative analysis through which we aim to know if there are differences in the averages of employee ratings depending on whether or not they mention certain advantages in their social marketing comments.

Table 1 shows the average ratings that employees give to companies depending on whether or not they mention in their comments the advantages related to the following dimensions (grouped advantages): social relations, working conditions, company image, benefits, facilities, and happiness. It is observed that when mentioning the advantages, the average score is higher, with the difference being more pronounced in the variables social relations (mean = 4.06), happiness (mean = 4.29), and company image (4.23). Another result worth highlighting is that the difference in the average evaluation of the benefits (depending on whether they are mentioned) is significant at a 90% confidence level. However, in this case, if it is not mentioned, the average rating is slightly higher than the average rating if it appears in the comments.

**Table 1:**
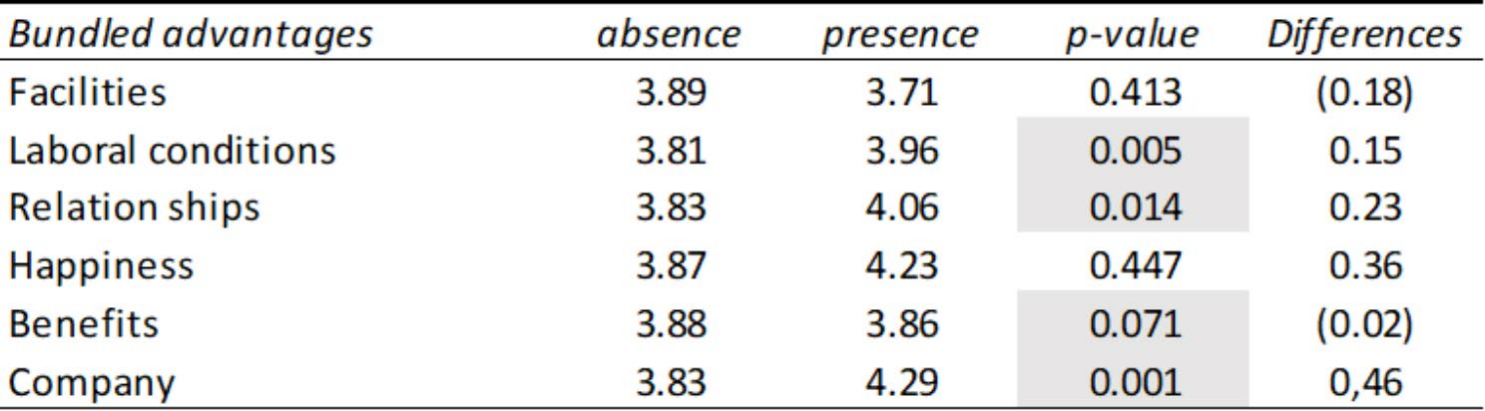
Summary table of means and T-Student corresponding to the presence or absence of grouped advantages in the comments linked to social marketing

*Source* Own elaboration

Once the dimensions most valued by employees had been verified, another T-Student test was carried out to verify which of the specific advantages are the ones that give rise to a more significant difference in the average rating depending on whether it is mentioned. or not in the comment. In this way, it can be seen in Table 2 that the following advantages with the most striking differences in evaluations are companion and company culture, in which there are significant differences depending on whether or not these advantages are mentioned in the comments.

**Table 2:**
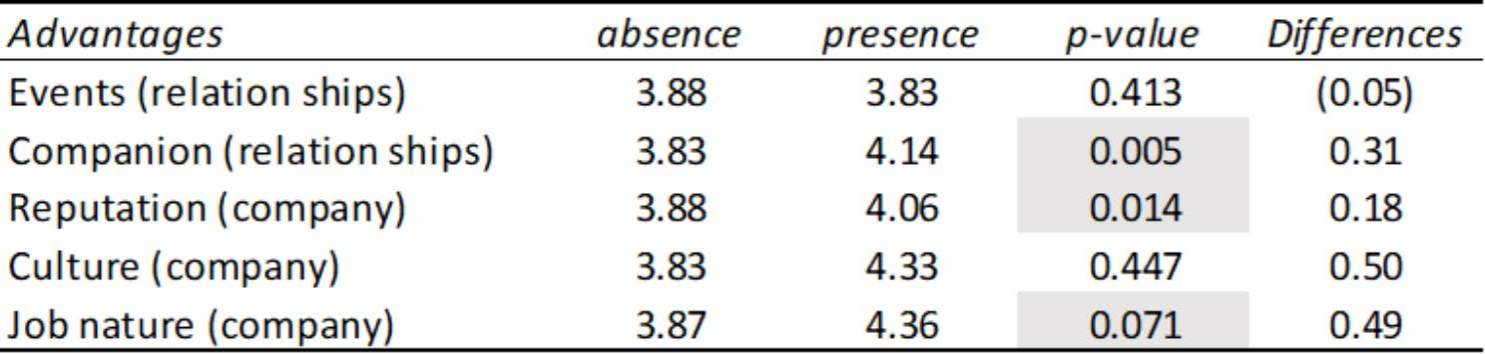
Summary table of means and T-Student corresponding to the presence or absence of advantages in comments linked to social marketing

*Source* Own elaboration

Finally, it has been verified whether there are significant differences in the average ratings that employees give by sector depending on whether they mention the advantages previously indicated in Table 3 in their comments. The results (Table 3) show that the differences observed in pairs (mention the advantage or not mentioning it) have been significant, according to the T-Student contrast at 95% confidence, in the following cases: Working conditions in the transportation sector, social relations in companies in the IT sector, and company image in the construction, consulting, and IT sectors. In the tourism sector, the difference in the average evaluations of benefits is significant at 90%.

**Table 3:**
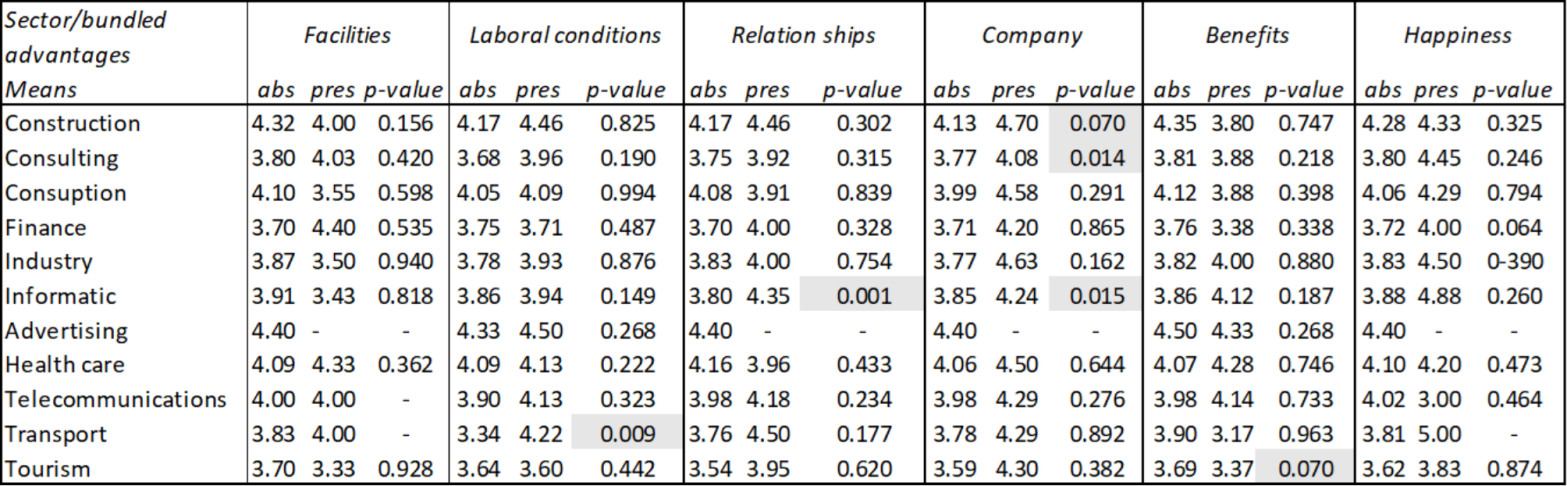
Summary table of averages and T-Student of advantages in social marketing comments by sectors

*Source* Own elaboration

**Fig. 1:**
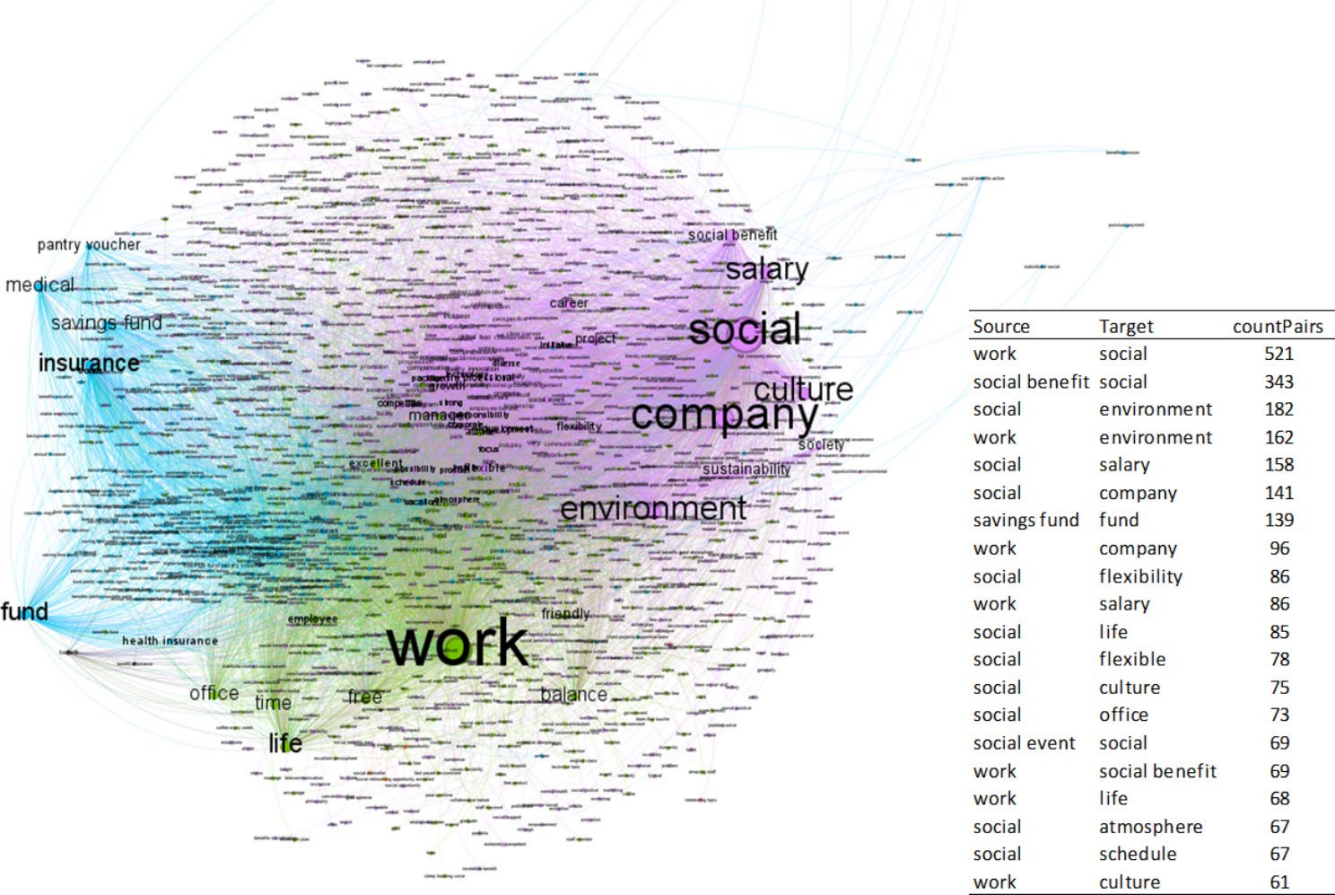
The semantic network of social marketing comments on advantages related to working conditions, corporate image, and social relations

Next, the semantic network generated from the comments on social marketing is presented (Fig. 1). Those that mention advantages related to working conditions, company image, and social relations have been considered because the differences between the average evaluations depending on whether or not the advantage is mentioned are significant.

Figure 1 shows the network generated from co-occurrences of words that appear in the same comment, made up of 1,300 nodes and 9,374 edges. In the framework, subgroups or communities are observed in networks established from the most frequent relationships between pairs of words. The nodes represent the words; the connections reflect whether they appear in the same comment. Depending on the modularity, each community is represented by a different color provided by the tool and represents a certain percentage of the network. The most representative communities and the terms that stand out the most are the following:

Community 1, in purple, represents 51.85% of the network. The terms social, business, and environment stand out in this community. The term social usually refers to activities of social responsibility and social action, including words such as society or community, as shown in this comment: “The culture you experience in Arup is truly aligned to the ethics and behavior lived by most leaders and employees. Diverse project opportunities that generate benefit to the community and broader society.” *(Arup*,* January 2022)*. Although the terms society or community indeed appear a considerable number on the Internet (58), they do not usually appear with other words recurrently in the comments.

Community 2, represented in green, represents 29.15% of the network. It includes work-related words like life, freedom, balance, and time. An example of this type of comment is one from Arup: “Good social events/perks and work-life balance. Friendly company culture.” (*Arup*,* July 2022*).

The third most representative community is the blue one, which represents 17% of the semantic network. In this group, words appear referring to issues such as health insurance or pension funds, as shown in this comment from Starbucks:

“Good benefits like health insurance, free tuition for an online university, the partner fund, and a long list of lots of other benefits. Free coffee, drinks, food and discounts at any Starbucks location were amazing, I never had to worry about where to get my next meal. Tips were good sometimes.” (*Starbucks*,* October 2022*).

### Results corresponding to comments related to Happiness Management

Table 4 represents how users link happiness with the dimensions of facilities, labor conditions, relationships, company, benefits and social marketing. That is, when users show characteristics related to happiness in their review, and at the same time they are mentioning some of these dimensions, the average rating of the review varies. To check whether the rating differences observed in the dimensions were statistically significant, a contrast of means for independent samples (t-student) was carried out.

It is observed that when dimensions such as facilities, relationships, company or benefits are mentioned, significantly different average ratings are produced, with the greatest differences occurring in the dimensions facilities (going from 4.15 to 3.71 on average), benefits (it went from 4.16 to 3.95 on average) and company (it went from 4.11 to 4.31 on average).

**Table 4:**
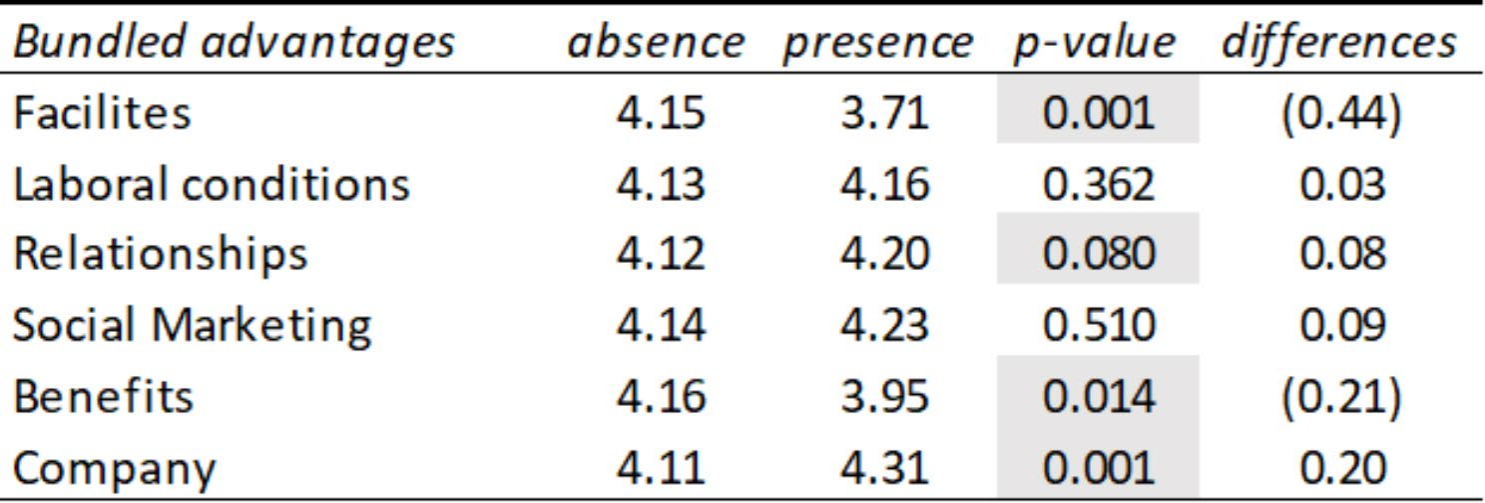
Summary table of means and T-Student corresponding to the presence or absence of advantages grouped in the comments linked to happiness

*Source* Own elaboration

Once we have found which dimensions are affected by happiness, we proceed to develop another contrast of means for independent samples that allows us to verify which variables or advantages of each dimension are those that give rise to the greatest differences in the evaluations or ratings. employee means. These differences are shown in Table 5.

**Table 5:**
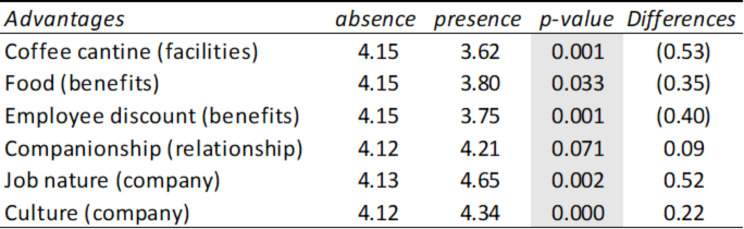
Summary table of means and T-Student corresponding to the presence or absence of advantages in comments linked to happiness

*Source* Own elaboration

It is observed that the greatest differences occur when characteristics of the coffee cantine are mentioned (it goes from 4.15 to 3.62), the job nature (it goes from 4.13 to 4.65), the employee’s discounts (from 4.15 to 3.75) and food (from 4.15 to 3.80).

**Table 6:**
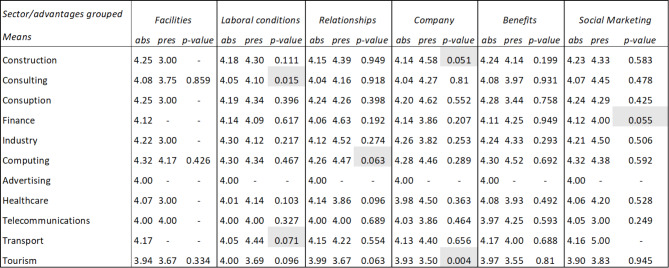
Summary table of averages and T-Student of advantages in comments from people referring to Happiness Management by sectors

*Source* Own elaboration

Table 6 represents how users from different sectors link happiness with the dimensions of facilities, labor conditions, relationships, company, benefits and social marketing. That is when users are showing characteristics related to happiness in their review, and at the same time they are mentioning some of these dimensions, the average rating of the review varies, and if this happens in each of the sectors. To check whether the rating differences observed in the dimensions were statistically significant, a contrast of means for independent samples (t-student) was carried out.

It is observed that when dimensions such as labor conditions, relationships, company or social marketing are mentioned, significantly different average ratings are obtained in some sectors.

Regarding labor conditions, different average ratings occur both in the consulting sector (it goes from 4.05 to 4.1) and in the transportation sector (it goes from 4.05 to 4.44), that is, while in transportation, when users mention aspects linked to labor conditions, this is reflected in an increase in their rating, in the consulting sector the opposite effect occurs, this aspect causes their rating to decrease.

In the computing sector, different average ratings also occur when attributes linked to relationships are mentioned (it goes from 4.26 to 4.47), therefore relationships are important in this sector. Furthermore, in the tourism sector, different average ratings are obtained when aspects of relationships are mentioned (it goes from 3.99 to 3.67), therefore relationships are less important in this sector. Finally, in the healthcare sector there are also significant differences (it goes from 4.14 to 3.86), therefore relationships are less important in this sector too.

Regarding the attributes linked to the company (such as its culture, mission, values, etc.), different average ratings are produced both in construction (it goes from 4.14 to 4.58) and in tourism (it goes from 3.93 to 3.50), that is, while in construction it implies a positive impact, increasing the rating, in tourism the opposite effect occurs, obtaining a lower rating. Finally, regarding social marketing attributes, different average ratings occur in the finance sector (it goes from 4.12 to 4.00), that is, it is not a valued aspect in this sector.

**Fig. 2:**
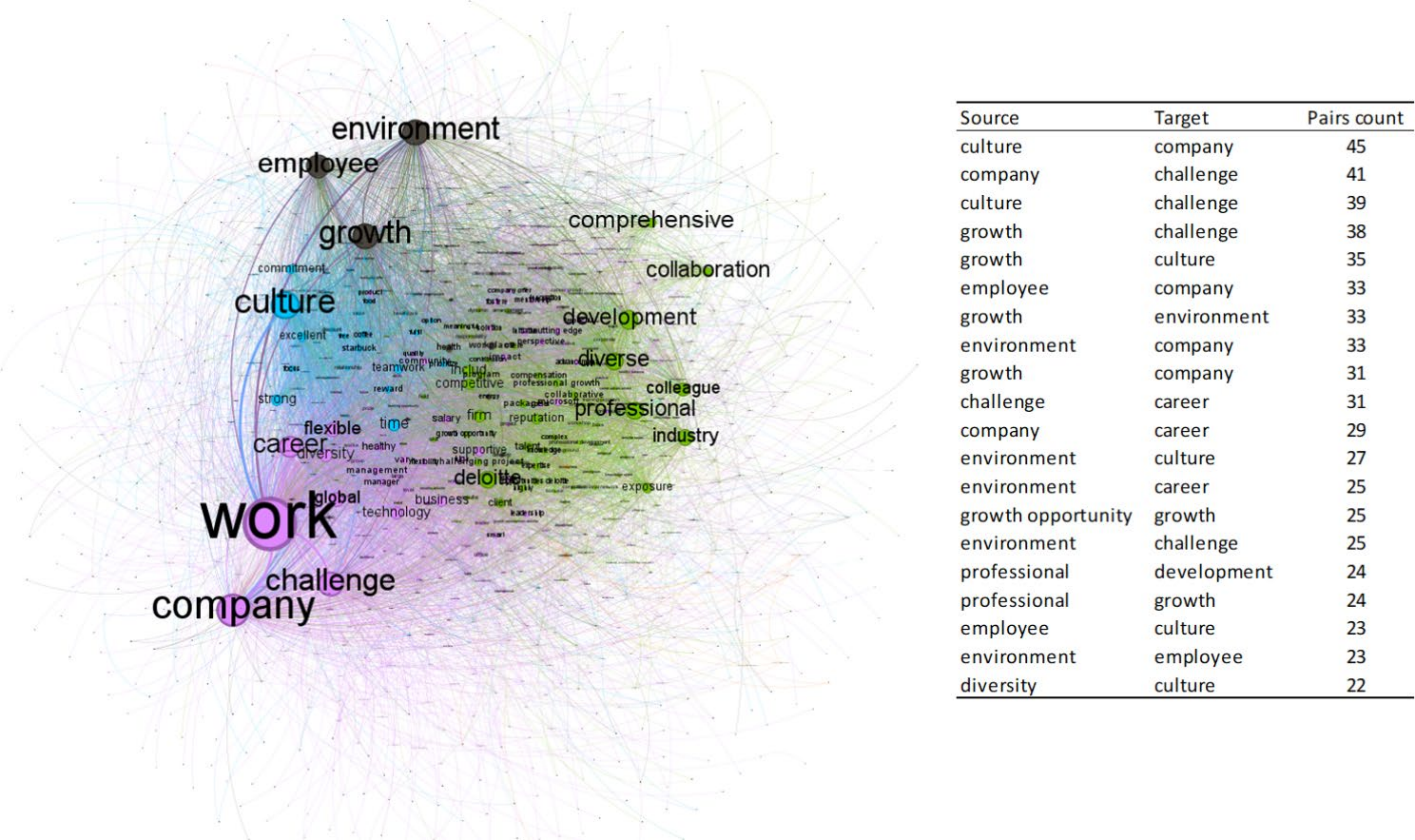
Semantic network of comments about happiness

Regarding the analysis of the network that represents the reviews that show happiness management in their comments, Fig. 2 shows the network generated from co-occurrences of words that appear in the same comment, formed by 906 nodes and 9,032 edges.

The most representative communities and the terms that stand out the most are the following:

Community 1, in purple, represents 47.35% of the network, that is, the words most connected to each other belong to this group. In this community the terms work, company, challenge, career and flexible stand out. The term challenge is mentioned by employees to express that companies give them opportunities to challenge their careers, through a challenging atmosphere. When the career is mentioned, it is in the context of career growth, advancement and progression. The term company is linked to both commitment and colleagues and collaboration. Finally, flexibility refers to a flexible environment that allows flexible work. An example of a review within this community is the following: “They train you constantly. If you are proactive, they give you room to grow and take on challenges. The teams are very collaborative. Good benefits.” (*Accenture*,* August 2023*).

Community 2, in green, represents 16.56% of the network. In this community, the terms comprehensive, collaboration, development, professional and colleague stand out. Employees mention the term comprehensive to express that companies facilitate comprehensive benefits. On the other hand, when collaboration is mentioned, it is in the context of a collaborative environment, with challenging projects and collaborative colleagues. The term development is linked to professional development. An example of a review within this community is the following: “Microsoft fosters a culture of innovation, offering numerous opportunities for career growth and skill development. The work environment encourages collaboration, and the company’s global impact is truly inspiring. The comprehensive benefits package and flexible work hours are definite advantages.” (*Microsoft*,* November*,* 2023*).

Community 3, in blue, represents 16.34% of the network. In this community, the terms culture, commitment, strong, time and teamwork stand out. The term culture is mentioned by employees to express that cultures related to challenge, competitiveness and collaboration are promoted in their companies. On the other hand, when commitment is mentioned, it is in the context of benefits, coffee and beverages. Another term that stands out is time, linked to free time and flexible time. Finally, teamwork is linked to strong work groups, which allow career development. An example of a review within this community is the following: “Great company culture, constant challenges, great teamwork, personal and professional growth, diversity and inclusion, balance between work and personal life.” (*HP Inc.*,* July 2021*).

Community 4, in grey, represents 16.23% of the network. In this community, the terms environment, employee and growth stand out. Employees mention the term growth to express growth opportunities. This term is also linked to employees since it identifies employees’ desire for employee growth, and with the term environment, since environments that encourage growth are rewarded. An example of a review within this community is the following: “The company inspires me with opportunity, growth, challenge and learning.” (*Amazon*,* November*,* 2021*).

Regarding the term work, it is the most mentioned in all the comments on this network about happiness. The most frequently mentioned terms are related to words such as culture, environment, challenge, growth career, flexibility, development, salary, leadership or reward (Table 7). Therefore, when employees show happiness with their jobs, they mention these terms.

**Table 7:** “Work” co-currence summary table

Word co-occurrence	count Pairs
work / culture	99
work / company	85
work / environment	83
work / challenge	77
work / career	51
work / growth	49
work / employee	43
work / flexible	32
work / professional	30
work / colleague	30
work / diverse	28
work / development	27
work / salary	25
work / supportive	25
work / leadership	24
work / excellent	24
work / vary	24
work / diversity	23
work / flexibility	22
work / technology	22
work / global	22
work / reward	21

*Source* Own elaboration

## Conclusions

This work reveals the relevance of employee opinions in the evaluations they give to companies from the perspective of Happiness Management and Social Marketing. Additionally, this research explores whether Glassdoor’s ranking of the best companies to work for provides meaningful information about which benefits employees value most. In this way, it would be possible to identify companies with the potential for superior performance in the future. For this, two types of analysis have been carried out, one quantitative and the other qualitative. The results of the quantitative analysis and those of the qualitative analysis have been differentiated into two blocks: the first corresponds to the comments referring to social marketing, and the second to those linked to happiness management. This distinction has been made because social marketing and happiness management are becoming increasingly important in the private sector. In this sense, it has been proven that when companies carry out social marketing campaigns, an improvement in their image is generated [53–55]. The company’s image is one of the critical factors in the results of the quantitative analysis in the first block since the mean differences in employee ratings are statistically significant depending on whether or not it is mentioned in the comments. Concerning the company’s image, issues related to reputation and culture are the most relevant. Canning et al. [59] also highlight employees’ importance to culture (also on the Glassdoor platform) in their research. These results are crucial as cultural norms are linked to greater employee trust and commitment [56].

Furthermore, reputation is another factor linked to the company’s image, which corresponds to other studies such as that of Shirin & Kleyn [61], who affirm that the company’s reputation positively influences its commitment. Working conditions and social relations have also been the advantages most valued by employees, along with the company’s corporate image, both in the social marketing and happiness comment blocks. However, in the block of social marketing comments, there are significant differences regarding specific advantages over social relationships, such as events and camaraderie. Camaraderie is revealed as a fundamental factor in employee comments on Glassdoor and in other studies such as that of Moro, Ramos, and Rita [56], whose results highlight that a positive attitude of co-workers contributes to a positive environment and improves job satisfaction.

In the transition to Industry 5.0, organizations increasingly recognize the critical role of employee happiness in driving innovation and competitive advantage. The literature review highlights the convergence of human-centered technology and automation, emphasizing not only the integration of advanced technologies but also the importance of fostering an environment conducive to creativity, ethics, and happiness [1]. This shift toward happiness economics suggests that employee well-being is directly related to organizational success, as happy employees tend to exhibit higher levels of productivity, creativity, and loyalty [2, 3].

The integration of Industry 5.0 human-centric technology into the workplace underlines a fundamental shift towards prioritizing employee happiness, as highlighted by Ravina-Ripoll et al. [9]. This transformation is not only technological but fundamentally human, where the happiness economy emerges as a cornerstone for organizational success [2, 3]. Quantitative analysis, particularly student t-tests, offers empirical validation of this relationship, highlighting how specific job benefits significantly influence employee happiness. This analysis echoes the findings of Lyubomirsky et al. [7], emphasizing the tangible impact of flexible work arrangements and health programs on employee satisfaction.

Analysis of employee reviews on the Glassdoor platform has provided valuable insights into the factors that influence job satisfaction and happiness. Glassdoor offers a candid look at organizational practices and their impact on the workforce, revealing a strong correlation between workplace benefits and employee happiness [5, 7]. Benefits such as flexible work arrangements, health and wellbeing programs and career development opportunities are increasingly valued in the changing Industry 5.0 workplace landscape, underscoring the need for organizations to align their benefits packages with the values and needs of their employees.

The relationship between social marketing and happiness management is critical in brand management, allowing brands to reflect and absorb societal culture [85]. In the business environment, a successful social marketing program must consider establishing explicit behavior change objectives, using the most appropriate communication channels, focusing on solid research on the target audience, paying attention to monitoring channels and evaluation, and developing a relational approach when addressing complex issues [86]. A model that addresses how to carry out effective social marketing management is the application of Rothschild’s MOA framework (Motivation, Opportunity, and Ability) [87]. In this sense, Binney, Hall, and Shaw [88] demonstrated an association between behavioral change and the constructs above. Going one step further, and due to the complexity of the behavior, Parkinson et al. [89] proposed adding one more construct to the MOA model, the nature of the behavior, which refers to the fact that the complexity of behavior is determined mainly by its inherent characteristics, and the levels of motivation, opportunity, and ability of the person, all of which will probably change over time. An adequate combination of Happiness management and social marketing can be addressed using the models and concepts mentioned above to improve social well-being.

The results of the qualitative analysis, through the semantic network, have allowed us to delve deeper into the context in which the comments appear, both in the social marketing and happiness blocks. Before continuing, it is necessary to highlight that semantic networks have been developed with the three most significant advantages according to the quantitative analysis: working conditions, social relations, and company image. In this sense, it stands out that in the largest community detected in the network of social marketing comments, mentions of valuable actions for society predominate, accentuating the importance of social marketing. This is also evident in other studies on employee comments on Glassdoor, such as that of LaVan, Zilic & Basappa [57], which measure the relevance of green jobs in companies. Another interesting result has been finding positive comments about the balance between work and family life. This fundamental aspect is also reflected in the research results by Hope et al. [58]. Regarding working conditions, it is worth highlighting the preponderance of aspects related to health insurance and pension funds; however, the differences in the average ratings have been insignificant in the previous T-Student analysis.

Qualitative analysis through semantic networks deepens the nuanced understanding of happiness within the workforce, revealing the complex interplay between job benefits and employee perceptions in different communities. Qualitative analysis conducted through semantic networks on comments extracted from Glassdoor offers a unique perspective on the complex fabric of employee happiness and satisfaction. This approach allows for a nuanced understanding of how various factors interact in the work environment, revealing the multifaceted nature of employee happiness beyond what quantitative measures can capture.

This approach, which explores the semantic relationships between key terms in employee reviews, reveals the subjective dimensions of happiness and satisfaction. It reveals how employees value not only tangible benefits but also the broader cultural and relational aspects of their workplace, aligning with the perspectives of Querbach et al. [5] on the importance of a supportive work environment.

The first community strongly emphasizes the importance of work-life balance and flexibility, reflecting a growing trend in employee expectations. The prominence of terms related to “life,” “freedom,” “balance,” and “time” indicates a significant valuation of policies that enable employees to harmonize their professional and personal lives. This finding aligns with research suggesting that today’s workforce appreciates and expects flexible work arrangements [5].

Another community focuses on health insurance and pension funds, highlighting the critical role of health and financial security in employee satisfaction. The debate around these benefits suggests that, despite the changing nature of work, traditional benefits remain critical to ensuring a sense of security and well-being among employees. Qualitative insights highlight the significant emphasis employees place on health insurance and pension funds, indicating the importance of health and financial security in fostering workplace satisfaction. This aligns with the findings of Luo et al. [13], who highlight the value that employees place on comprehensive health benefits, linking them to greater job satisfaction and general well-being. The emphasis on health and well-being benefits in employee comments reflects broader trends in the literature, suggesting that such benefits are crucial to ensuring a sense of security and well-being among employees. Additionally, qualitative analysis supports the idea that, despite the changing nature of work, traditional benefits, such as health insurance, remain critical to improving employee satisfaction [13].

Another community focuses on professional growth, challenges and professional development opportunities. The frequent mention of “challenge”, “growth”, “career” and “professional development” underlines the importance that employees place on being provided avenues to advance and develop within their roles. These findings align with the work of Sekar et al. [30], who found that positive sentiments in online reviews significantly affect overall employee satisfaction, with skill development being a strong predictor of satisfaction. The emphasis on career challenges and growth opportunities reflects a critical aspect of employee engagement and satisfaction, suggesting that the workforce highly values career development opportunities. This is further corroborated by the analysis presented in their paper, which highlights the importance of providing employees with pathways to advance and develop in their roles, thereby improving job satisfaction and promoting a culture of continuous learning and improvement [32].

The latter community reveals a deep commitment to the company’s culture, values and collaborative environment. Terms like “culture,” “engagement,” “teamwork,” and “collaboration” suggest that employees highly value a positive, inclusive, and supportive work culture. This is consistent with literature indicating that organizational culture significantly impacts job satisfaction and employee retention [2, 3].

## Theoretical and managerial implications

### Theoretical implications

The analysis of worker opinions on Glassdoor offers rich theoretical implications for academic literature, particularly in the realms of happiness management, the happiness economy in Industry 5.0, and social marketing. These implications extend across various disciplines, providing a nuanced understanding of employee satisfaction and its broader impacts on organizational success and societal well-being. These platforms, by providing unfiltered insights into employee experiences, satisfaction, and well-being, serve as a valuable resource for understanding the dynamics of workplace happiness and its broader economic and social implications.

In the context of happiness management, employee Glassdoor reviews can significantly contribute to the development of theories related to job satisfaction and employee well-being. Reviews often highlight factors that contribute to or detract from workplace happiness, such as leadership quality, work-life balance, and recognition [90]. By analyzing these inputs, researchers can refine models of happiness management, emphasizing the importance of psychological well-being in the workplace [91]. This analysis can lead to a better understanding of how positive work environments foster not only higher productivity but also greater employee loyalty and creativity [92]. For instance, studies have shown that job satisfaction is linked to reduced employee turnover and increased productivity [93]. By analyzing employee reviews, researchers can identify specific aspects of work-life that impact happiness, such as leadership quality, work-life balance, recognition, and career development opportunities. This analysis can enrich the theoretical frameworks of happiness management by providing empirical evidence of how these factors are perceived by employees across different industries and organizational contexts.

Employee reviews on Glassdoor also have implications for social marketing, particularly in how organizations communicate their values and culture to attract talent and engage with broader societal issues. Reviews act as a form of electronic word-of-mouth (eWOM) that can influence employer branding and corporate reputation, thus, positive employee feedback can be a powerful tool for attracting talent, building brand loyalty, and enhancing corporate reputation. Conversely, negative reviews can highlight areas where organizations need to improve their social responsibility efforts, such as promoting diversity, equity, and inclusion, or implementing environmentally sustainable practices. Analyzing these reviews allows for a deeper understanding of the social constructs that drive job choice and employee engagement, offering insights into how companies can leverage social marketing strategies to promote a positive work environment and corporate social responsibility. This analysis can inform theories on how effective social marketing can enhance employee satisfaction and happiness, thereby contributing to a positive organizational image and attracting socially conscious consumers and employees. This feedback loop between employee satisfaction and corporate social marketing strategies underscores the importance of internal stakeholders in shaping a company’s external image. It also provides a basis for developing theories on the role of employee happiness in the broader context of corporate social responsibility and ethical business practices [94].

Furthermore, this research is aligned with different studies and allows the existing gap to be reduced in three ways. First, this research addresses the need to explore the value of online employee reviews from various perspectives, leveraging big data to gain a competitive advantage and improve companies’ strategies [31]. Secondly, it deepens the study of the benefits that companies offer their employees, not only in one sector but in a wide range of industries [39]. Thirdly, an analysis differentiated by sectors and dimensions of benefits has been carried out, covering a research need that several authors called for [13, 41].

### Managerial implications

Analyzing the opinions left by workers on Glassdoor is important for several compelling reasons, impacting both organizations and the broader job market. This platform offers a treasure trove of candid feedback from current and former employees about their work experiences, company culture, management practices, and job satisfaction. This feedback is invaluable for multiple stakeholders, including job seekers, company management, and researchers.

For job seekers, these reviews provide insights into the company’s working conditions, growth opportunities, and the overall satisfaction levels of its employees [8]. This information helps them make informed decisions about whether a company aligns with their career goals and personal values [13]. It essentially allows them to look beyond the glossy exterior presented by corporate marketing and get a glimpse of the real working environment. Social marketing is presented as a powerful tool to promote behavioral changes aimed at improving individual well-being and that of the community in general. The application of social marketing in the workplace is essential, especially when it comes to promoting a culture that values happiness, collaborative participation, adaptability and innovation [42, 45]. The results of this research highlight how social marketing strategies, by addressing barriers and leveraging motivations, can lead to sustainable behavioral changes, including improving employee satisfaction and creating a positive work environment.

From a company’s perspective, employee reviews on Glassdoor can serve as a powerful tool for self-assessment and improvement. By analyzing these reviews, companies can identify areas of strength and areas needing improvement [11]. This could range from enhancing communication and leadership practices to revising compensation and benefits packages [15]. Addressing these issues not only helps in attracting top talent but also in retaining current employees by showing that the company values their feedback and is committed to creating a positive work environment. Analysis of employee reviews on Glassdoor reveals that happiness management and social marketing are intrinsically linked to the perception of an organization’s culture and values. Positive feedback about the work environment, culture and benefits contributes significantly to a company’s image, attracting and retaining talent. Employee ratings, and the benefits they mention in their reviews, are the result of effective social marketing, which not only improves job satisfaction but also promotes a sense of belonging and community among employees, which are key drivers of happiness. in the workplace. Additionally, this research explores how social marketing can amplify the visibility of an organization’s commitment to innovative benefits, work-life balance, and a supportive work atmosphere. By effectively communicating these values, organizations can reinforce their reputation as reputable employers among workers, thereby attracting candidates who share similar values and are more likely to experience satisfaction and happiness at work.

Moreover, for researchers and analysts, these reviews offer a rich dataset for studying employment trends, job satisfaction determinants, and the impact of corporate culture on employee well-being [7]. They can use this data to identify patterns and correlations that can inform broader discussions on work-life balance, employee engagement, and organizational effectiveness [5]. To maximize the impact on employee happiness and organizational performance, companies must strategically integrate happiness management initiatives with social marketing strategies. This involves not only providing meaningful benefits and fostering a positive work culture but also effectively communicating these efforts both internally and externally. By doing so, organizations can create a feedback loop in which increased employee happiness generates positive reviews and testimonials, further improving the company’s image through social marketing.

This research contributes to happiness management theories by empirically demonstrating how positive job environments enhance productivity, loyalty, and creativity. These insights show how leadership quality, work-life balance, and recognition contribute to workplace happiness, enhancing productivity, loyalty, and creativity. Such feedback aids job seekers in making informed decisions, helps companies improve practices and attract talent, and provides researchers with valuable data on employment trends and corporate culture’s effect on employee well-being.

By analyzing employee reviews on Glassdoor, organizations can gain valuable insights into the factors that contribute to job satisfaction and happiness. Furthermore, understanding and addressing the evolving needs of their workforce allows companies to leverage job benefits as a powerful tool for fostering a happy, productive, and innovative workplace.

Despite the wealth of information available, there remains a paucity of empirical studies that fully leverage this real-time data. This study addresses this gap by utilizing Glassdoor reviews to explore employee satisfaction, demonstrating the value of these digital platforms in capturing the fluid and evolving nature of workplace happiness.

Furthermore, for job seekers, these reviews provide insights into companies’ working conditions, growth opportunities, and overall satisfaction levels. From a company’s perspective, employee reviews on Glassdoor can serve as a powerful tool for self-assessment and improvement. For researchers and analysts, these reviews offer a rich dataset for studying employment trends, job satisfaction determinants, and the impact of corporate culture on employee well-being.

These practical implications underscore the importance of leveraging the feedback employees share on Glassdoor to inform both happiness management and social marketing strategies. By understanding and addressing the needs and preferences of their workforce, organizations can improve job satisfaction, employee well-being, and ultimately, organizational performance. This holistic approach aligns with the broader goals of Industry 5.0, which emphasize the value of integrating human-centered practices with technological advances to foster a sustainable, innovative and happy workplace.

## Limitations and future research lines

Using user opinions on Glassdoor to analyze happiness management has limitations. Firstly, the self-selection bias means the reviews may not represent the entire workforce, as typically those with extreme opinions are more inclined to post, which is why this research has only analyzed the benefits highlighted by employees, and not the disadvantages. Glassdoor also allows knowing these negative reviews, so a future line of research would be to focus on the negative aspects that workers highlight.

Secondly, anonymity can lead to exaggerated positive or negative feedback, impacting the reliability of the data. However, this research has initially downloaded 138,765 employee reviews, which makes it possible to reduce this possible bias.

Additionally, the lack of demographic and job-specific information limits the ability to contextualize responses or identify trends across different employee groups. Glassdoor allows knowing the country, city and job position of each person who publishes a review, so a future line of research should replicate this analysis taking into account the geographical diversity and the types of benefits that are applied in each country [38].

The self-selection bias inherent in Glassdoor reviews means that the data may disproportionately represent employees with extreme positive or negative experiences. To mitigate this, we included a large sample size of 138,764 reviews, which helps to balance out individual biases. Additionally, the anonymity of the reviews can lead to exaggerated feedback, both positive and negative. By focusing on consistent keywords and patterns across many reviews, we aimed to derive more reliable insights.

Another limitation is the lack of demographic data, such as age, gender, and job position, which restricts our ability to analyze trends across different employee groups. While we ensured diversity by including companies from various sectors, future research could benefit from incorporating demographic information to provide more nuanced insights. Lastly, reviews reflect perceptions at a specific point in time and may not capture ongoing changes within organizations or industry trends. Collecting reviews over a three-year span (2021–2023) helped to mitigate this by capturing more stable trends and reducing the impact of short-term fluctuations.

Finally, these reviews reflect individual perceptions at a point in time and may not accurately capture ongoing changes within the organization or industry trends. Glassdoor also allows knowing the date on which the review is published, so future research could analyze the evolution of the benefits offered by companies over time, or for example the differences before, during and after COVID-19.

Concerning the concept of the happiness economy within Industry 5.0, Glassdoor employee reviews can shed light on how the adoption of Industry 5.0 technologies—such as artificial intelligence, robotics, and the Internet of Things—affects employee happiness and job satisfaction. For example, research could explore how automation and digitalization impact workers’ sense of job security, skill development opportunities, and work satisfaction. The feedback from employees can provide insights into the balance between technological efficiency and human well-being, contributing to the development of a happiness economy that values both productivity and employee satisfaction. This aligns with the broader goals of Industry 5.0, which focus on creating a sustainable and human-centric industry [95].

Analyzing Glassdoor worker opinions extends across disciplines, offering a holistic view of the interconnectedness of employee happiness, organizational success, and social well-being. These reviews provide empirical data that can enrich theories in happiness management by detailing the specific workplace factors that contribute to or detract from employee well-being. In the context of Industry 5.0, they offer insights into the human side of technological advancement, highlighting the importance of designing technologies and work processes that enhance, rather than undermine, human happiness. For social marketing, these reviews underscore the role of authentic employee voices in shaping organizational brands and driving social change.

## Data Availability

The datasets used and/or analysed during the current study are available from the corresponding author on reasonable request.
